# Advanced Magnetic Resonance Imaging in Pediatric Glioblastomas

**DOI:** 10.3389/fneur.2021.733323

**Published:** 2021-11-10

**Authors:** Fabrício Guimarães Gonçalves, Angela N. Viaene, Arastoo Vossough

**Affiliations:** ^1^Division of Neuroradiology, Department of Radiology, Children's Hospital of Philadelphia, Philadelphia, PA, United States; ^2^Department of Pathology and Laboratory Medicine, Children's Hospital of Philadelphia, Philadelphia, PA, United States; ^3^Department of Pathology and Laboratory Medicine, Perelman School of Medicine, University of Pennsylvania, Philadelphia, PA, United States; ^4^Department of Radiology, Perelman School of Medicine, University of Pennsylvania, Philadelphia, PA, United States

**Keywords:** advanced MRI, children, conventional MRI, diffusion-weighted imaging, glioblastoma, magnetic resonance spectroscopy, perfusion weighted imaging

## Abstract

The shortly upcoming 5th edition of the World Health Organization Classification of Tumors of the Central Nervous System is bringing extensive changes in the terminology of diffuse high-grade gliomas (DHGGs). Previously “glioblastoma,” as a descriptive entity, could have been applied to classify some tumors from the family of pediatric or adult DHGGs. However, now the term “glioblastoma” has been divested and is no longer applied to tumors in the family of pediatric types of DHGGs. As an entity, glioblastoma remains, however, in the family of adult types of diffuse gliomas under the insignia of “glioblastoma, IDH-wildtype.” Of note, glioblastomas still can be detected in children when glioblastoma, IDH-wildtype is found in this population, despite being much more common in adults. Despite the separation from the family of pediatric types of DHGGs, what was previously labeled as “pediatric glioblastomas” still remains with novel labels and as new entities. As a result of advances in molecular biology, most of the previously called “pediatric glioblastomas” are now classified in one of the four family members of pediatric types of DHGGs. In this review, the term glioblastoma is still apocryphally employed mainly due to its historical relevance and the paucity of recent literature dealing with the recently described new entities. Therefore, “glioblastoma” is used here as an umbrella term in the attempt to encompass multiple entities such as astrocytoma, IDH-mutant (grade 4); glioblastoma, IDH-wildtype; diffuse hemispheric glioma, H3 G34-mutant; diffuse pediatric-type high-grade glioma, H3-wildtype and IDH-wildtype; and high grade infant-type hemispheric glioma. Glioblastomas are highly aggressive neoplasms. They may arise anywhere in the developing central nervous system, including the spinal cord. Signs and symptoms are non-specific, typically of short duration, and usually derived from increased intracranial pressure or seizure. Localized symptoms may also occur. The standard of care of “pediatric glioblastomas” is not well-established, typically composed of surgery with maximal safe tumor resection. Subsequent chemoradiation is recommended if the patient is older than 3 years. If younger than 3 years, surgery is followed by chemotherapy. In general, “pediatric glioblastomas” also have a poor prognosis despite surgery and adjuvant therapy. Magnetic resonance imaging (MRI) is the imaging modality of choice for the evaluation of glioblastomas. In addition to the typical conventional MRI features, i.e., highly heterogeneous invasive masses with indistinct borders, mass effect on surrounding structures, and a variable degree of enhancement, the lesions may show restricted diffusion in the solid components, hemorrhage, and increased perfusion, reflecting increased vascularity and angiogenesis. In addition, magnetic resonance spectroscopy has proven helpful in pre- and postsurgical evaluation. Lastly, we will refer to new MRI techniques, which have already been applied in evaluating adult glioblastomas, with promising results, yet not widely utilized in children.

## Introduction

This review was conceived and developed during a watershed moment, in which major changes are occurring in how brain tumors are classified. During the last few years, new concepts and entities have emerged, and well-known diagnoses and terminology have been abandoned. According to the shortly upcoming 5th edition of the World Health Organization Classification of Tumors of the Central Nervous System (CNS) (WHO/CNS/5), the term “glioblastoma” is no longer applicable for tumors in the family of pediatric types of diffuse high-grade gliomas (DHGGs) (1). Glioblastoma only remains, as an entity, in the family of adult types of diffuse gliomas (1).

As stated in the WHO/CNS/5, there are three components in the family of adult types of diffuse gliomas and four in the family of pediatric types of DHGG. The three adult types of diffuse gliomas are represented by the (1) astrocytoma, IDH-mutant (grades 2, 3, and 4); (2) oligodendroglioma, IDH-mutant, and 1p/19q-codeleted (grades 2 and 3) and (3) glioblastoma, IDH-wildtype (grade 4) (1). The four pediatric types of DHGGs (all grade 4 tumors) are represented by the (1) diffuse midline glioma, H3 K27-altered; (2) diffuse hemispheric glioma, H3 G34-mutant; (3) diffuse pediatric-type high-grade glioma, H3-wildtype and IDH-wildtype; and (4) infant-type hemispheric glioma (1). This review will focus on the pediatric types of DHGGs (except the diffuse midline glioma, H3 K27-altered) and the two grade 4 adult types of diffuse gliomas, namely the astrocytoma, IDH-mutant grade 4 (formerly known as glioblastoma, IDH-mutant), and the glioblastoma, IDH-wildtype (1). These two latter diffuse gliomas are typically found in adults, but can also occasionally occur in older children, particularly teenagers.

Despite the separation and removal of the term “glioblastoma” from the family of pediatric type DHGGs, as per the WHO/CNS/5 (1), this paper will still apocryphally employ the term due to a number reasons, including: (1) its preeminent historical relevance, (2) the bulk of the available neuro-oncology literature refers to it as glioblastoma, (3) the paucity of recent literature explicitly dealing with the most current classification (WHO/CNS/5), and (4) a convenient way to refer to grade 4 DHGGs that can occur in children, beyond the diffuse midline glioma, H3 K27-altered, and glioblastoma, IDH-wildtype, which may still be sometimes seen in the pediatric population.

Studies on glioblastomas in children are more limited in number than their adult counterparts, despite this tumor's clinical relevance and the substantial patient burden that accompanies this diagnosis. Even though “glioblastomas in children” share similar morphological characteristics to adult glioblastomas, they are much less common and present distinct gene expression and molecular profiles, explaining observed differences in adjuvant treatment response. Given their relative rarity, most of the available literature relies on studies involving small numbers of patients in the form of case reports and small case series. Fundamental concepts of pathology, clinical features, management, structural imaging, and advanced imaging techniques (some not widely available such as metabolic and physiologic magnetic resonance imaging) to study “glioblastomas in children” will be reviewed.

## Background

Glioblastomas are highly aggressive tumors whose cell of origin is not fully clarified. They are the most lethal and most common primary CNS neoplasm in adults, with incidence peak in the sixth and seventh decades (2). They are relatively rare in children, representing 0.6–7.9% of all “glioblastomas” (3), and accounting for 3–15% of all primary pediatric CNS tumors (4). They can occur at any age, more frequently around the second decade of life (4–6).

“Glioblastomas” may arise anywhere in the developing CNS. Their most common location is in the supratentorial compartment (7), occurring in 30–50% of the patients in the cerebral hemispheres (8). Involvement of deeper structures such as the thalamus, corpus callosum, and hypothalamus is less common (9). Spinal cord involvement is rare, representing only 3% of all cases (8). Very rarely “pediatric glioblastomas” may occur in the cerebellum (1–2% of all patients) (10).

### Signs and Symptoms

Signs and symptoms are often non-specific, typically of short duration, and usually result from increased intracranial pressure (headache, behavior changes, early morning nausea/emesis, diplopia, papilledema, and altered sensorium) (4, 5, 11). In addition, localizing symptoms may occur, namely focal motor deficits, hemiplegia, pyramidal tract findings, dysmetria, and chorea (12). Infants and young children may also manifest ambiguously with failure to thrive, lethargy, and macrocephaly. Finally, precipitous neurological deterioration may also occur, commonly from intratumoral hemorrhage or seizures (9). Seizures may be present in around 30% of affected children, more commonly when lesions are superficially located in the frontal or temporal lobes (5, 11, 13).

### Pathology and Molecular Diagnosis

**Figure 1:**
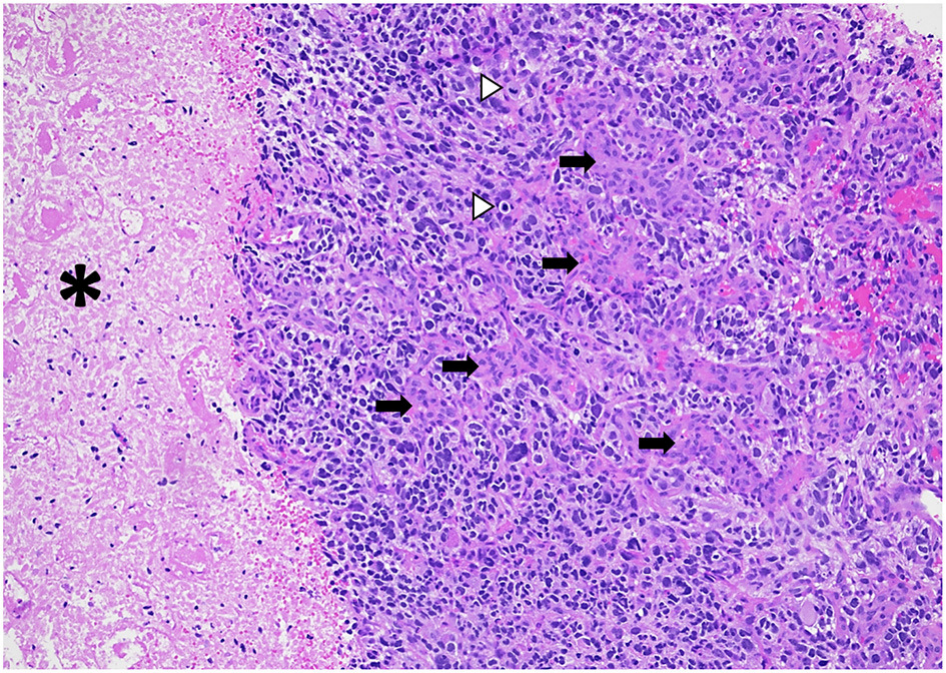
Diffuse hemispheric glioblastoma, H3 G34 mutant (histologic “glioblastoma”) demonstrating high cellularity, nuclear pleomorphism, microvascular proliferation (arrows), necrosis (asterisks), and mitotic figures (arrowheads). HandE stain, 100x magnification.

“Glioblastomas” are typically large, highly vascularized, heterogeneous, and infiltrative masses, and often have irregular margins. On gross pathology, the peripheral rims are pink-gray and solid and may contain a yellow, soft necrotic center, and often contain hemorrhagic foci (14). Microscopically, they typically show increased cellularity, high mitotic activity, pleomorphic cells, microvascular proliferation, and necrosis (Figure 1). There are no gross or microscopic histologic differences between “glioblastomas” affecting adults or children (15–17); however, certain histologic subtypes may be more frequently encountered in specific age groups.

**Figure 2:**
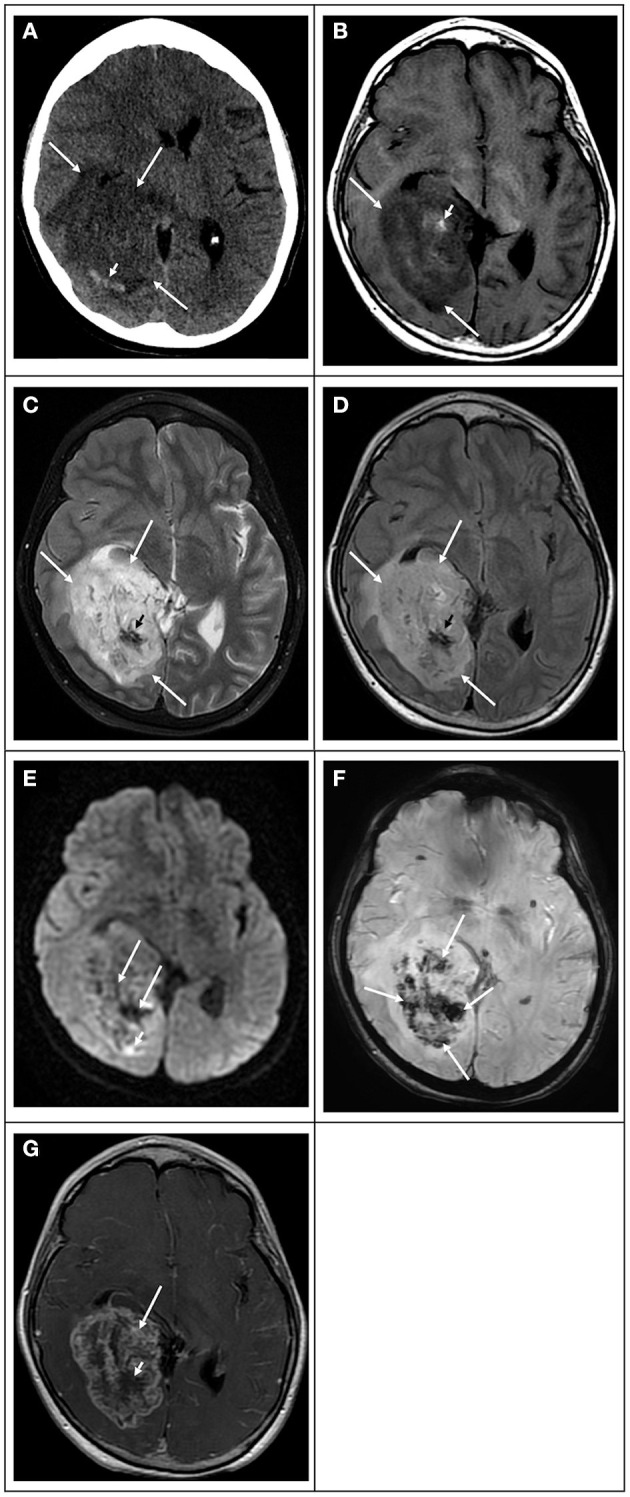
Radiation-induced secondary “glioblastoma” in a 17-year-old girl who had a previous posterior fossa ependymoma at age 5. **(A)** Computed tomography image in the axial plane shows a large ill-defined tumor in the right temporal and occipital lobes (white arrows), causing mass effect, compression of the right lateral ventricle, and midline deviation. The lesion is associated with vasogenic edema and hemorrhage (white arrowhead). **(B)** T1 weighted image in the axial plane shows that the bulk of the lesion is hypointense (white arrows) with scattered hyperintensity foci due to intratumoral hemorrhage (white arrowhead). **(C)** T2 weighted image and **(D)** FLAIR image in the axial plane show that the bulk of the lesion is hyperintense with scattered hypointense foci due to intratumoral hemorrhage. **(E)** Diffusion-weighted image in the axial plane shows that the lesion is heterogeneous in signal. The bulk of the lesion is isointense to the normal brain parenchyma with scattered hyperintense foci due to intratumoral hemorrhage and small peripheral hyperintense foci (white arrowhead), which had lower values on the ADC maps (not shown) in keeping with restricted diffusion. **(F)** Susceptibility weighted image in the axial plane shows extensive signal drop within the tumoral bed (white arrows) in keeping with diffuse hemorrhage. **(G)** T1-weighted contrast-enhanced image in the axial plane shows that the tumor enhances heterogeneously. The mass has avid enhancement with central non-enhancing areas in keeping with necrotic tissue.

Their diffusely infiltrative behavior is characteristic, typically showing hypercellularity, nuclear atypia, necrosis (which may be pseudopalisading), and vascular endothelial cell proliferation. Glioblastomas are friable and highly vascularized masses in which thrombosed vessels, bleeding, and necrosis can be identified (18). Calcification is atypical but can be seen in radiation-associated “secondary glioblastomas.” Radiation-associated “secondary glioblastomas” may be observed many years following CNS radiation treatment for childhood malignancies, including leukemia and other brain tumors such as medulloblastomas and ependymomas (Figure 2) (18). However, radiation-associated “secondary glioblastomas” are uncommon, with an average time to develop around 9 years (18). Contrary to adult patients, the progression of low-grade gliomas into “secondary glioblastoma” is very rare in children.

**Figure 3:**
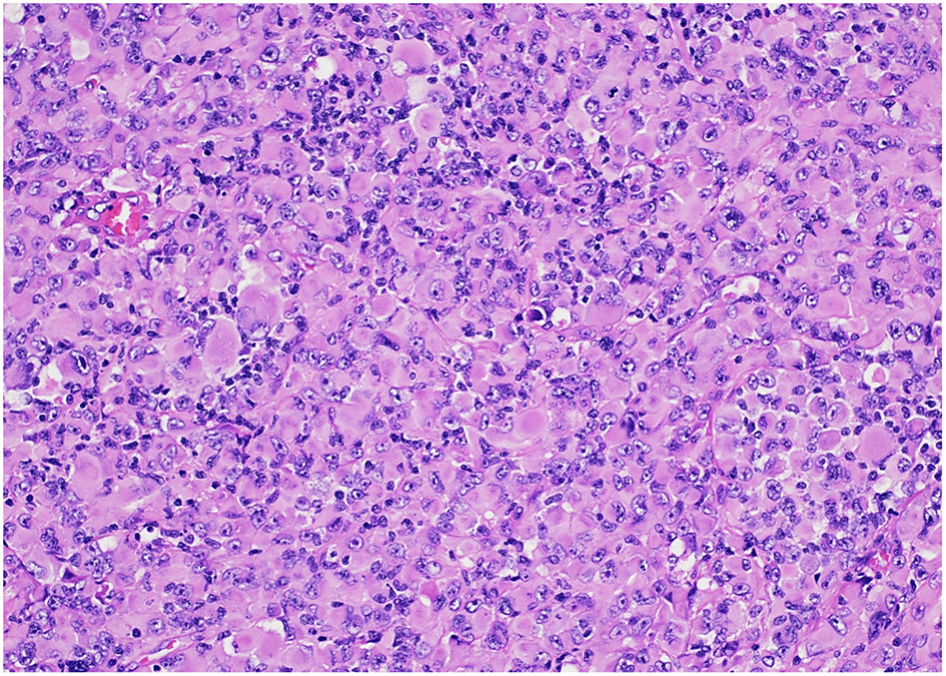
Diffuse pediatric-type high-grade glioma, H3-wildtype and IDH-wildtype (“histologic epithelioid glioblastoma”) characterized by sheets of tumor cells with distinct nuclear borders, a moderate to large amount of eosinophilic cytoplasm, eccentrically-located nuclei, and prominent nucleoli. HandE stain, 200x magnification.

Traditionally, glioblastomas have been histologically classified as giant-cell glioblastoma, gliosarcoma, and epithelioid glioblastoma (19). Epithelioid “glioblastomas” are known to be more common in children and are characterized by large eosinophilic cells, prominent melanoma-like nuclei, and often rhabdoid cells (Figure 3) (9). However, in the WHO/CNS/5, subtypes are not listed in the classification, but are further discussed in their respective chapters (1).

Many high-grade gliomas (HGGs) have also more recently been classified and diagnosed by methylation profiling, and entities once thought to comprise “pediatric glioblastomas” have been reclassified as other entities and vice versa. In addition, molecular features may vary by location. For example, what was thought to be a uniform group of glioblastomas occurring in the posterior fossa, now comprises distinct molecular entities (based on methylation profiles), namely anaplastic astrocytoma with piloid features; glioblastoma, IDH wildtype; diffuse midline glioma H3 K27M mutant; and astrocytoma, IDH mutant (20).

Previously, CNS tumor grading fundamentally relied on histological features with rare exceptions (i.e., diffuse midline glioma, H3K27M-mutant). Currently, specific molecular markers also play a major role in diagnosis and prognosis and even in assignment of tumor grade. The WHO/CNS/5 has combined histological and molecular grading, meaning that molecular parameters have now been added as biomarkers of grading and can sometimes upgrade a lesion, despite its conventional histology characteristics (1).

Formerly, glioblastomas have been classified, based on the isocitrate dehydrogenase (IDH) gene mutation status, into three major subgroups: IDH-wildtype (the majority of the patients), IDH-mutant, and not otherwise specified (19, 21). “IDH-mutant glioblastomas” were often considered secondary neoplasms (i.e., progressed from lower-grade IDH-mutant astrocytomas), while IDH-wildtype glioblastomas typically represented *de novo* neoplasms, mainly in older adults (9).

According to WHO/CNS/5, glioblastomas, as valid entities, are, by definition, only represented by the IDH-wildtype. They may also incorporate three genetic parameters, namely TERT promoter mutation, EGFR gene amplification, the combined gain of entire chromosome 7, and loss of entire chromosome 10 [+7/−10] (1). Therefore, the presence of one or more of the latter three in a patient with IDH-wildtype diffuse astrocytic tumor suffices to assign the highest WHO grade (1). In the family of adult diffuse gliomas, the tumors that do not show microvascular proliferation or necrosis, but show either TERT promoter mutation, EGFR mutation, or +7/-10 chromosome copy number changes will still be classified as glioblastoma when considering the integrated diagnosis (1). In other words, even though there may not be full histologic features of “glioblastoma” in these diffuse gliomas, the addition of specific molecular information may still classify them as glioblastoma. Note that occasionally, adult type glioblastoma, IDH-wild type may be seen in older children.

At present, the WHO/CNS/5 (1) recognizes three adult types of diffuse gliomas, namely:

1) Astrocytoma, IDH-mutant
2) Oligodendroglioma, IDH-mutant, and 1p/19q-codeleted and the
3) Glioblastoma, IDH-wildtype.

Astrocytomas, IDH-mutant, can be classified in grades 2, 3, or 4 (formerly IDH-mutant glioblastoma) (1). Notably, the term “glioblastoma” is no longer valid in the setting of a pediatric type diffuse glioma (1). In addition, the WHO/CNS/5 (1) endorses four pediatric types of DHHGs, namely:

1) Diffuse midline glioma, H3 K27-altered
2) Diffuse hemispheric glioma, H3 G34-mutant
3) Diffuse pediatric-type high-grade glioma, H3-wildtype and IDH-wildtype
4) Infant-type hemispheric glioma.

**Figure 4:**
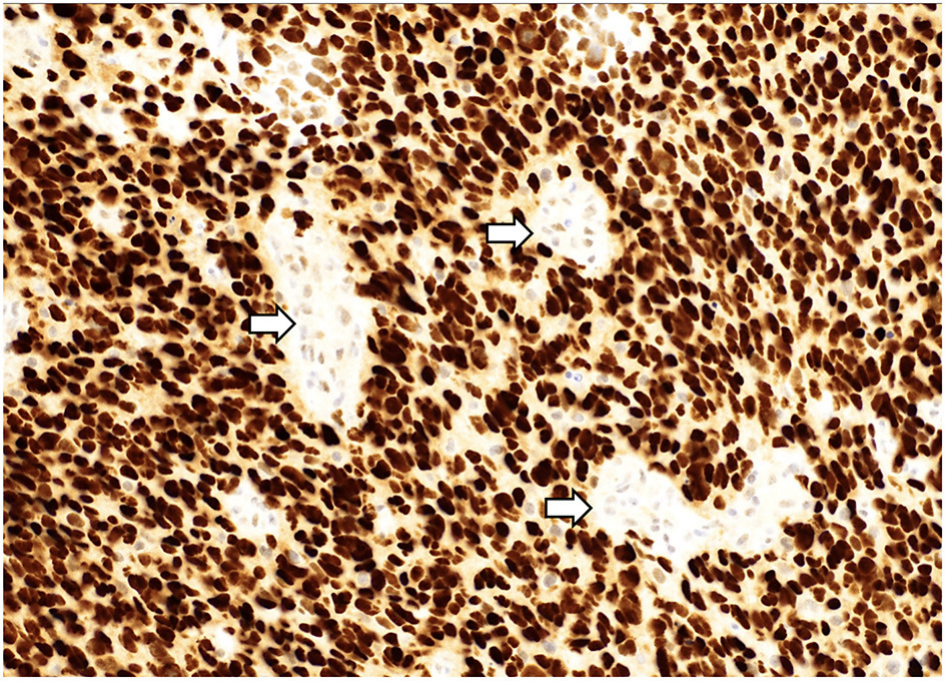
A “glioblastoma” with a mutation in TP53 demonstrating strong, diffuse nuclear staining for p53 with wildtype staining present in vessels (arrows). p53 immunostain, 200x magnification.

The characteristically altered genes and molecular profiles in each of the four pediatric types of DHHGs are (1):

1) Diffuse midline glioma, H3 K27-altered: H3 K27, TP53, ACVR1, PDGFRA, EGFR, EZHIP
2) Diffuse hemispheric glioma, H3 G34-mutant: H3 G34, TP53 (Figure 4), ATRX
3) Diffuse pediatric-type high-grade glioma, H3-wildtype, and IDH-wildtype: IDH-wildtype, H3-wildtype, PDGFRA, MYCN, EGFR (methylome)
4) Infant-type hemispheric glioma: NTRK family, ALK, ROS, MET.

Lack of IDH mutation is known to impact therapy outcomes negatively in adult patients. “Glioblastomas in children” and other HGG typically demonstrate a low incidence of IDH mutation (seen in ~6% of all pediatric HGGs), and such alterations are infrequent in younger children (22). Therefore, the majority of true glioblastomas in children are thought to be IDH-wildtype. The incidence of IDH-mutant or “secondary glioblastomas” may be higher in older adolescents and younger adults (23).

**Figure 5:**
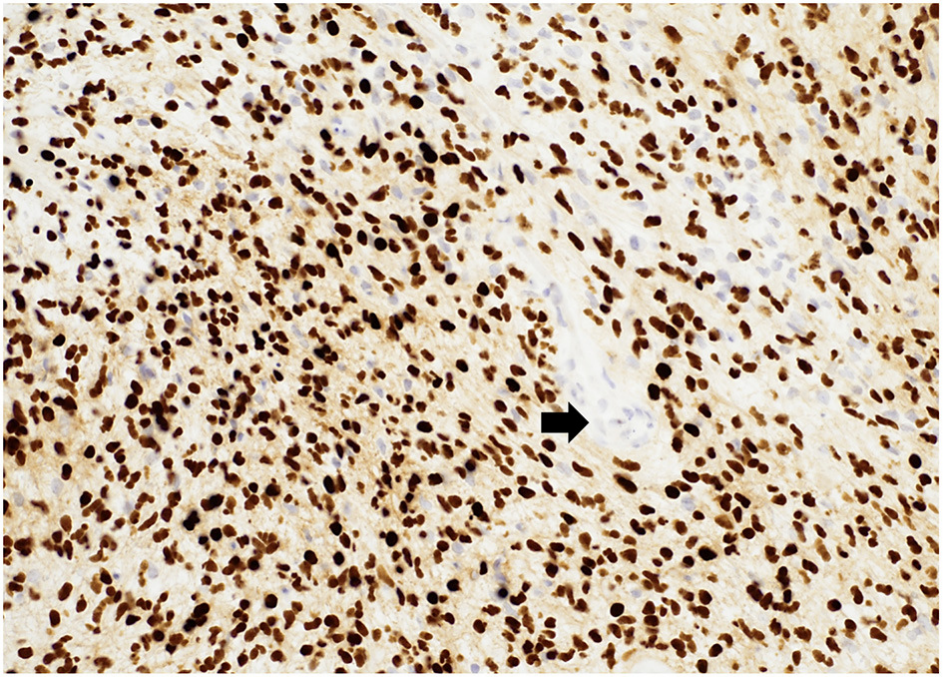
Diffuse midline glioma, H3K27M-altered demonstrating diffuse nuclear staining for H3K27M with negative endothelial cells serving as an internal control (arrow). H3K27M immunostain, 200x magnification.

**Figure 6:**
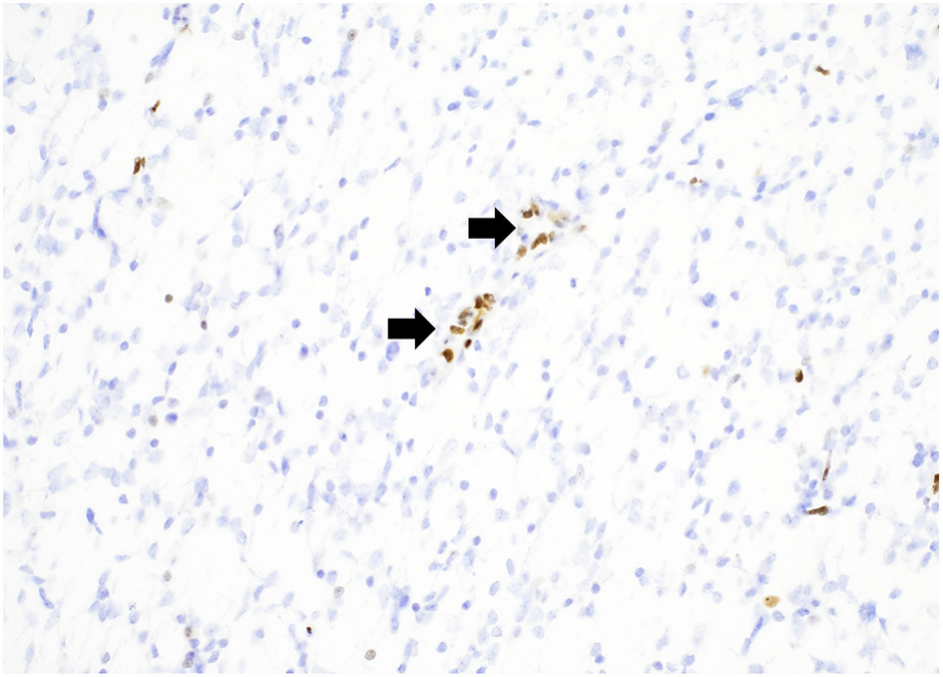
Diffuse midline glioma, H3K27-altered demonstrating a loss of nuclear staining for H3K27me3 with positive endothelial cells serving as an internal control (arrows). H3K27me3 immunostain, 200x magnification.

**Figure 7:**
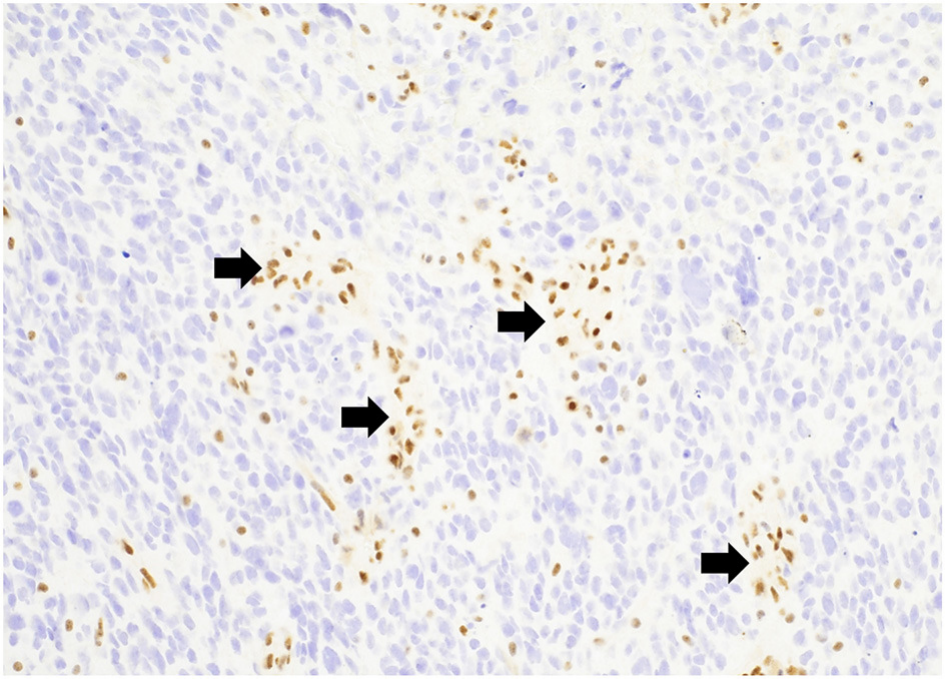
A “glioblastoma” with an ATRX mutation demonstrating a loss of nuclear staining for ATRX with positive endothelial cells serving as an internal control (arrows). ATRX immunostain, 200x magnification.

Mutations in histone genes are the most common molecular findings in pediatric HGGs with H3 K27M mutations associated with midline tumors (Figures 5, 6) and H3 p.G34R/V mutations occurring in hemispheric tumors. The majority of these tumors are histologically high-grade; however, it should be noted that infiltrative, astrocytic tumors of the midline with H3 K27M mutations are classified as “diffuse midline gliomas,” and regardless of histologic grade, correspond to WHO grade 4 (19). In addition, co-occurring mutations in TP53 (Figure 4) and ATRX (Figure 7) may be seen in tumors with H3 mutations, whereas IDH mutations are not observed (24, 25). Of note, circumscribed/non-diffuse gliomas of the midline are not considered grade 4, according to the (WHO/CNS/5) (26). Diffuse midline gliomas are not the primary focus of this paper.

Diffuse midline gliomas are one of the most severe pediatric brain tumors, with dismal prognosis despite developments in diagnosis and therapeutics. According to Castel et al. (27) there are two subgroups of diffuse midline gliomas, H3-K27M-mutant, namely H3.1-K27M and H3.3-K27M with differences in prognosis and phenotypes. According to them, the differences between H3.1/H3.3 subgroups may be a result of distinct cells of origin or due to the type of histone mutated. In their study they found that the type of histone H3 mutated could also predict the outcome of DIPG patients more efficiently than clinical and radiological characteristics of the tumors (27). An in depth discussion about diffuse midline gliomas was not included here because of their complexity and specificities and due to space limitations, being necessary a specific paper to discuss them accordingly.

“Pediatric glioblastomas” commonly have a higher incidence of p53 mutation/overexpression (particularly in children <3 years) than mutation of epithelial growth factor receptor (EGFR) or deletion of phosphatase and tensin homolog (PTEN), which are common features of “adult glioblastomas” (9). In addition, ATRX mutations (Figure 7) have been reported in a fraction of “pediatric glioblastomas,” usually associated with other mutations (28). Vascular endothelial growth factor (VEGF) is commonly expressed by “adult glioblastomas” and is responsible for increased vascularity, tumor progression, and infiltration. Therefore, anti-VEGF (bevacizumab) therapy is frequently employed in “adult glioblastomas.” However, VEGF expression is relatively infrequent in “pediatric glioblastomas,” which may explain the comparative ineffectiveness of anti-VEGF therapy in children (29).

The mechanism of action of temozolomide (TMZ), one of the chemotherapy agents to treat glioblastomas, is by promoting DNA methylation. On the other hand, O^6^-Methylguanine-DNA Methyltransferase (MGMT) is a DNA repair enzyme, rescuing neoplastic cells from alkylating agent-induced damage. Therefore, MGMT activation leads to increased resistance to chemotherapy with alkylating agents. MGMT promoter methylation status has crucial prognostic importance in glioblastomas. Inactivation of MGMT generally correlates with chemotherapy responsiveness (30) and increased median event-free survival (31). Studies on MGMT expression in “pediatric glioblastomas” have demonstrated little alteration in the methylation promoter status in children, which may explain the reduced efficacy of TMZ in children compared to adults (32). Regarding the diffuse midline gliomas, H3 K27M mutant, MGMT promoter is unmethylated in almost all cases, which explains the failure of clinical trials administrating TMZ to patients with this diagnosis (33, 34). Whenever present, the prognostic significance of inactivating hypermethylation of MGMT confers a survival benefit to affected children.

## Neuroimaging

### Computed Tomography

**Figure 8:**
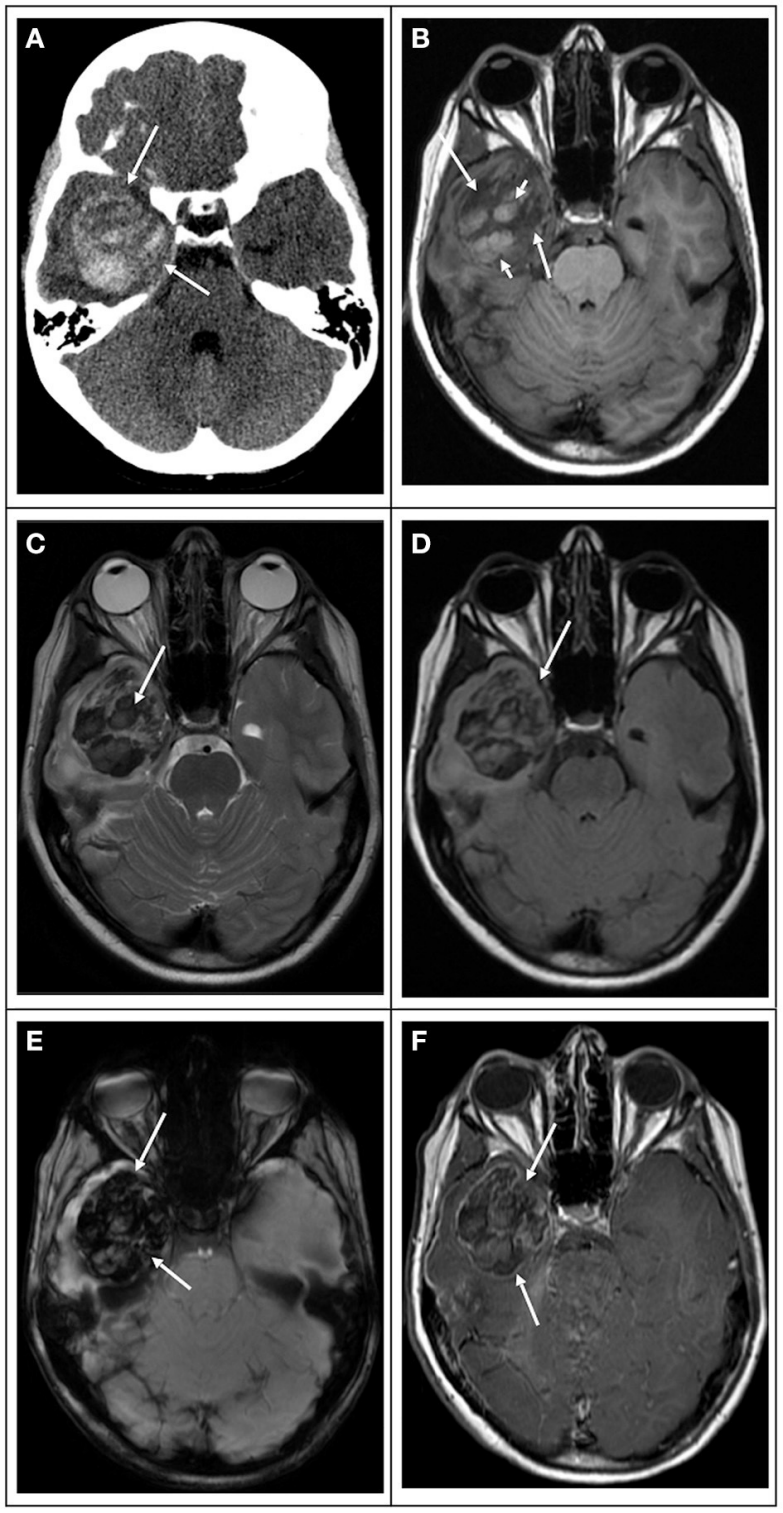
10-year-old male with worsening headaches and papilledema due to a right temporal lobe “glioblastoma”. **(A)** Computed tomography image in the axial plane shows a heterogeneously hyperdense area in the right temporal lobe in keeping with a diffusely hemorrhagic lesion. At this point, the differential includes vascular lesions or tumors. **(B)** T1 weighted image in the axial plane shows that the lesion is heterogeneous with mixed signals. The tumoral bed is hypointense (white arrows) with scattered hyperintensity components due to intratumoral hemorrhage (white arrowheads). **(C)** T2 weighted image and **(D)** FLAIR images in the axial plane show that the bulk of the lesion is hypointense due to diffuse intratumoral hemorrhage. **(E)** Susceptibility weighted image in the axial plane shows extensive signal drop within the tumoral bed (white arrows) in keeping with diffuse hemorrhage. **(F)** T1-weighted contrast-enhanced image in the axial plane shows that the tumor enhances heterogeneously. The mass has thin peripheral enhancement (arrowhead) with internal variable minimal enhancement due to hemorrhage and necrosis.

Computed tomography (CT) may be the first imaging modality in children to detect an intracranial neoplasm. On CT, “pediatric glioblastomas” typically present as poorly marginated heterogeneous lesions with mass effect and variable areas of hyperattenuation, which may be partially due to hemorrhage (Figure 8). Areas of hypoattenuation may correspond to necrosis or surrounding edema. Contrast-enhanced CT features are variable, ranging from minor to marked enhancement and from solid to heterogeneous enhancement. Necrotic lesions may show rim enhancement, typically with irregular borders (35). Typical CT findings of “pediatric glioblastomas” can be seen in the Table 1.

**Table 1:** Typical computed tomography and magnetic resonance imaging features of pediatric glioblastomas.

**Imaging features**	
Generalities	- Variable size (typically large) - Poorly marginated - Heterogeneous - Mass effect - Variable hemorrhage - Variable vasogenic edema
Location	- Cerebral hemispheres (most common) - Brainstem - Cerebellum - Spinal cord
**Computed tomography**	
Non-enhanced	- Variable areas of hyperattenuation (hemorrhage/hypercellularity) - Hypoattenuation → necrosis or surrounding edema
	
Contrast enhanced	- Variable enhancement - Minor to marked - Solid to heterogeneous - Necrotic lesions may present ring enhancement
**Magnetic resonance imaging**	
T1WI	- Iso- to hypointense (relative to gray matter) - Edema and necrosis → Hypointense
T2WI	- Hyperintense (relative to gray matter) - Variable vasogenic edema → hyperintense
FLAIR	- Hyperintense (relative to gray matter) - Variable vasogenic edema → hyperintense
Contrast enhanced T1WI	-Variable - Complex - Thick irregular rim (typical) - No enhancement (rare)
Susceptibility weighted imaging and T2^*^ weighted imaging	- When neoplasms are complicated by hemorrhage → low signal
Diffusion weighted imaging	- Variable restricted diffusion - Restricted diffusion (typical in solid areas)
Dynamic susceptibility contrast	- ↑ CBV
Dynamic contrast-enhanced	- ↑ K^trans^ - ↑ K^ep^ - ↑ Ve - ↑ CBV - ↑ CBF
Arterial spin labeling	- ↑ CBF
Proton 1/H Magnetic resonance	- ↑ Choline
spectroscopy	- ↓ NAA - ↑ Lactate

*T1WI, T1 Weighted imaging/T2WI, T2 Weighted Imaging. ↑, high; ↓, low; →, resulting in*.

### Magnetic Resonance Imaging

Magnetic Resonance Imaging (MRI) is the imaging modality of choice in the evaluation of “pediatric glioblastomas.” It delivers superior spatial and contrast resolution, allowing improved non-invasive assessment of the tumor and surrounding brain, and helps with neurosurgical and radiation planning (36). MRI findings of “pediatric glioblastomas” are not specific. Masses are typically heterogeneous with indistinct margins, mass effect on surrounding structures, and a variable degree of enhancement (complex, variable, or rarely absent). Relative to gray matter, “pediatric glioblastomas” may demonstrate iso- to hypointense T1 signal and heterogeneously hyperintense T2 signal with surrounding edema, which is readily evident on fluid attenuation inversion recovery (FLAIR) images (35, 37). When the neoplasms are complicated by hemorrhage, different signal characteristics can be seen, such as T1 hyperintense, T2 hypointense, low signal on T2^*^, and susceptibility-weighted imaging. Typical conventional MRI features of “pediatric glioblastomas” can be seen in the Table 1.

A distinction must be made between contrast enhancement and increased perfusion. The degree and extent of contrast enhancement reflect pathologic changes of the blood-brain barrier and extravascular leakage of contrast (38, 39). The blood-brain barrier breakdown can result from the destruction of normal capillaries by a neoplastic process or from the pathologic structure of the vascular walls of newly formed abnormal capillaries. The degree of perfusion reflects tumor vascularity, which may or may not be associated with blood-brain barrier breakdown (38, 39).

“Pediatric glioblastomas” are known to be less common than adult glioblastomas. As stated previously, there are no gross and microscopic pathology differences between adult and pediatric glioblastomas. However, there are major molecular and genetic differences between, which is unequivocally demonstrated and established by the WHO/CNS/5. In addition, these tumors also differ in terms of cause-specific survival and overall survival (9).

Literature comparing imaging differences between adult and pediatric glioblastomas is very scarce. Both tumors are typically heterogeneous and may demonstrate mass effect, vasogenic edema, restricted diffusion, and contrast enhancement. “Pediatric glioblastomas” may present as masses with more solid enhancement, whereas adult glioblastomas have a higher tendency to be necrotic, and therefore more commonly present as rim enhancing lesions.

### Advanced MRI Neuroimaging

The number of studies and evidence supporting the utility of advanced MRI neuroimaging techniques in children is much more limited than in adult glioblastomas, but the general principles similarly apply. Most of the literature on advanced MRI neuroimaging techniques in children is with limited affirmed clinical validation if analyzed in isolation. Thus, these techniques should be applied in conjunction with conventional imaging, under a multiparametric approach, for the imaging evaluation of brain tumor diagnosis and follow-up.

#### Diffusion-Weighted Imaging

Diffusion-weighted imaging (DWI) is a well-known clinical imaging technique applied in the study of brain tumors. However, there are very few studies dedicated specifically to using diffusion-weighted imaging (DWI) and apparent diffusion coefficient (ADC) measurements in assessing “pediatric glioblastomas.” DWI is a superb technique that measures the degree of movement of water molecules and probes their relation to the surrounding environment in both the normal and diseased states. Quantitative DWI information on the extent of the movement of water molecules can be obtained by the apparent ADC calculation (40, 41).

DWI is a mainstay in routine brain tumor MRI, typically acquired with two b-values for ADC calculation (b = 0 and b = 1,000 s/mm^2^). DWI is very helpful in pointing toward the diagnosis, providing information regarding tumor grade and type, and monitoring treatment response (42). In addition, several authors have shown that the different components of the tumors, surrounding edema, and normal surrounding white matter may have distinct ADC values (40, 43–45).

Several authors have demonstrated the utility of DWI in the differential diagnosis of cystic masses (46–49). Cystic and necrotic components of a tumor have higher ADC values, reflecting the increased water movement characterizing these components. Enhancing components of HGGs typically show lower ADC values than non-enhancing tumor and peritumoral edema (40). DWI often demonstrates restricted (reduced) diffusion (low ADC values) in solid portions of HGGs, in keeping with high cellular density and/or high nuclear-to-cytoplasm ratios (50). Restricted diffusion can be observed in some gliomas, notably higher grade tumors such as anaplastic astrocytomas, glioblastomas, diffuse midline gliomas, and anaplastic ependymomas.

DWI has been used to distinguish areas of peritumoral neoplastic cell infiltration from peritumoral edema, a critical distinction when dealing with HGGs (40). In addition, ADC values can be used to distinguish normal white matter from necrotic or cystic areas, edema, and solid-enhancing tumors (51). However, the distinction between infiltrating tumors from edema or nearby normal-appearing brain is sometimes inaccurate in glioblastoma, as small amounts of infiltrating tumor cells may be present in these areas, but not in enough quantities to significantly alter the diffusion parameters. DWI may also aid in the differentiation of abscesses from necrotic or cystic brain tumors such as HGGs (52, 53).

ADC is negatively correlated with cell proliferation indices such as Ki-67 (54). The signal characteristics on DWI and ADC maps can be strongly correlated to grade in pediatric brain tumors, and they may assist with preoperative diagnostic predictions (55). In addition, ADC measurements can be used to differentiate between HGGs from low-grade gliomas. Wang et al. (56), in a recent meta-analysis including 1,172 patients, found an area under the curve (AUC) for *b* values of 1,000 and 3,000 s/mm^2^ to be of 0.91 and 0.92, respectively. Their results demonstrated that ADC measurements had high diagnostic performance in discriminating HGGs from low-grade gliomas.

A study by Chang et al. (35) involving 11 patients with glioblastomas found that the DWI signal intensity in the solid portion of the tumor was hyperintense compared to the white matter. The ADC values for the solid tumor component ranged from 0.53 to 1.30 × 10^−3^ mm^2^/s (mean, 1.011 ± 0.29 × 10^−3^ mm^2^/s) and for white matter from 0.60 to 0.98 × 10^−3^ mm^2^/s (mean, 0.824 ± 0.130 × 10^−3^ mm^2^/s). In one of the patients, a recurrent glioblastoma demonstrated increased DWI signal in the tumoral bed months after total gross removal of the tumor (35). Tumor recurrence has been reported to have significantly lower ADC values than radiation necrosis (57, 58). DWI and ADC calculation may be utilized as a surrogate marker for monitoring the response of tumor therapy (59, 60). For example, Chenevert et al. (59) found a rapid increase in the mean ADC values shortly after treatment initiation, and the magnitude of the diffusion changes corresponded with clinical outcome.

Most published experience in applying DWI in pediatric tumors has focused mainly on posterior fossa tumors. Higher ADC values at the baseline have been reported to have a more favorable outcome in patients of diffuse midline glioma (61–63). In addition, baseline ADC values can be used as an outcome predictor in these tumors, although diffusion-derived metrics showed no significant association with overall survival (64).

#### Diffusion Tensor Imaging

Literature specifically dealing with diffusion-tensor imaging (DTI) in assessing “pediatric glioblastomas” is quite scarce. DTI is a more sophisticated quantitative analysis of diffusion-based imaging. In DTI, water movement is measured in several directions within the tissue from which tensors can be fit with directionality information. When these measurements are combined and analyzed, in addition to an averaged measure of water diffusion for each voxel (ADC), individual and summary measures of how water diffusion varies along different axes (fraction anisotropy—FA) can be calculated. Both ADC and FA reflect the microstructure of the tissue in which they are measured (65). For example, FA estimates the amount and the direction of diffusion restriction of water molecules along myelinated white matter tracts, which is in turn partly influenced by the degree of preserved and destroyed white matter tracts within the tumoral area (66–68).

**Figure 9:**
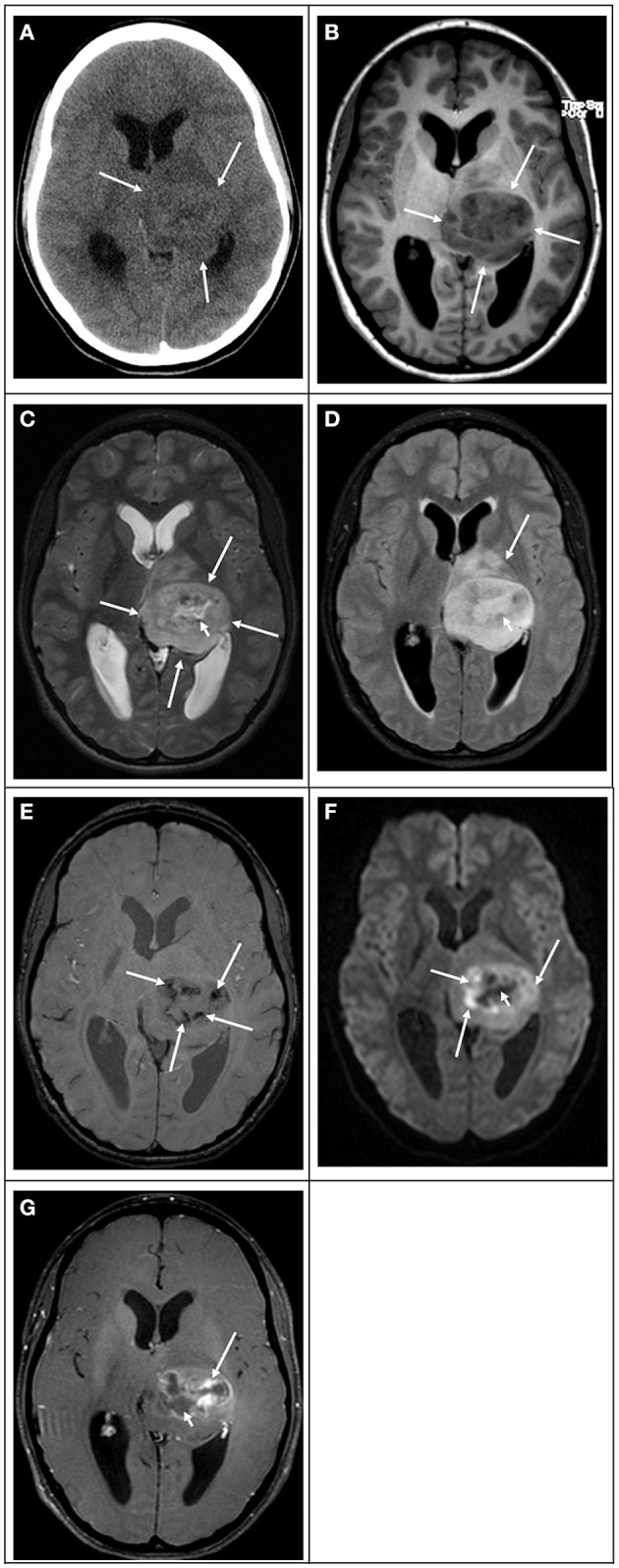
13-year-old female with blurry vision, facial weakness, and right-sided paresthesias due to a left thalamic glioblastoma. **(A)** Computed tomography image in the axial plane shows an ill-defined slightly hyperdense mass in the left thalamus. **(B)** T1 weighted image in the axial plane shows that the lesion is well-demarcated with heterogeneous mixed-signal, predominantly hypointense (white arrows). **(C)** T2 weighted image and **(D)** FLAIR images in the axial plane show that the bulk of the lesion is hyperintense (white arrows) with a central component showing even higher signal. **(E)** Susceptibility weighted image in the axial plane shows irregular signal drop foci within the tumoral bed (white arrows) in keeping with hemorrhage. **(F)** Diffusion-weighted image in the axial plane shows that the lesion is heterogeneous in signal. The periphery of the mass is isointense to the normal brain parenchyma. The center of the mass shows a hypointense signal (white arrow). Surrounding the center, there is an irregular hyperintense thick rim with low values in the ADC map (not shown) in keeping with restricted diffusion. **(G)** T1-weighted contrast-enhanced image in the axial plane shows that the tumor enhances heterogeneously. The thick irregular area that shows restricted diffusion enhances avidly (white arrows) with no enhancement centrally due to necrosis.

DTI can demonstrate white matter tracts and their structural changes related to different brain pathologies. DTI describes the three-dimensional diffusion phenomenon of water molecules about their microenvironmental properties allowing a unique description of the space where this molecular movement occurs. This model provides an *in-vivo* demonstration of the complex ultrastructural organization of the white matter and structural changes due to tumor invasion. Current DTI and magnetic resonance tractography applications allow accurate graphic delineation of the eloquent white matter tracts and their relation with tumoral tissue, which may be essential in surgical treatment planning and useful in assessing post-therapeutic changes (69). Furthermore, DTI in mapping white matter tracts may sometimes enable resection of tumors previously deemed unresectable, such as well-defined pilocytic astrocytomas in the thalamus (Figure 9) (70).

Several authors demonstrated that the evaluation of the peritumoral area and the differentiation between tumoral infiltration and pure vasogenic edema might be enhanced by DTI analysis (68, 71–76). Other authors quantified tumoral and peritumoral FA in low- and high-grade gliomas with low-grade gliomas showing higher FA values than HGGs (76–80). The more conspicuous FA reduction in high-grade compared to low-grade tumors is likely due to the higher incidence of cystic and necrotic changes leading to loss of white matter tract organization and integrity. Besides, some researchers also suggest that FA is more sensitive than ADC in the early detection of white matter tumoral involvement (67, 68, 78). White matter tract changes can occur due to fiber destruction (reduced absolute number), fiber edema (reduced density and higher water content), or fiber degradation (abnormal fibers with a normal number and density) (81, 82). In addition, qualitative assessment of DTI maps can suggest the different types of tumoral involvement such as displacement, edema, infiltration, destruction, or a combination of two or more, but one has to be careful of the technical limitations that may lead to false-negative tract visualization (68, 83–85).

Gauvain et al. (65) demonstrated the utility of ADC obtained via DTI assessing pediatric brain tumors. ADC correlated significantly with tumor cellularity and the calculated total nuclear area (nuclear area of each tumor cell type multiplied by the number of cells per high-power field). Brunberg et al. (51) found differences between ADC and FA from normal white matter and solid-enhancing tumor, cystic and necrotic areas, and regions of edema. However, they did not find differences in ADC values between various glioma subtypes.

DTI is also valuable in the assessment of postoperative changes. For example, DTI can compare the status of eloquent cortical pathways before and after the surgery delivering information to the neurosurgeon about the damaged and preserved tracts postoperatively (86–88). DTI also allows detecting and monitoring treatment-induced neurotoxicity in cerebral white matter (87, 89–92). Several studies investigating the effect of cranial irradiation and chemotherapy indicate a prominent decrease in mean FA values that are more severe in the frontal lobes compared with the parietal lobes despite the same radiation dose, suggesting regional susceptibility in the frontal lobe (89–94). Mabbott et al. (93) demonstrated that medulloblastomas treated with cranial-spinal radiation therapy show abnormal FA and ADC in the normal-appearing white matter. Their study observed damaged white matter microstructure and/or fiber integrity as demonstrated by FA and ADC for multiple regions within the cerebral hemispheres. Further, decreased FA and increased ADC were related to lower intellectual outcomes in patients relative to age-matched controls. A significant advantage of DTI is that it provides measures sensitive to underlying tissue properties, and hence potential damage may be evident even within the normal-appearing white matter.

### Perfusion Weighted Imaging

Perfusion weighted imaging (PWI) describes a group of valuable techniques that can non-invasively evaluate the cerebral hemodynamic status and, in many patients, can predict tumor grade and behavior (35, 51). The main clinically utilized PWI techniques include dynamic contrast-enhanced (DCE), dynamic susceptibility contrast (DSC), and arterial spin labeling (ASL). The main parameters derived from these techniques include cerebral blood volume (CBV), cerebral blood flow (CBF), and mean transit time (MTT), among others. Derivation of absolute perfusion parameters may be challenging at times, and therefore, relative CBV (rCBV) or relative CBF (rCBF) may be used when involved regions are normalized to other normal-appearing brain structures.

**Figure 10:**
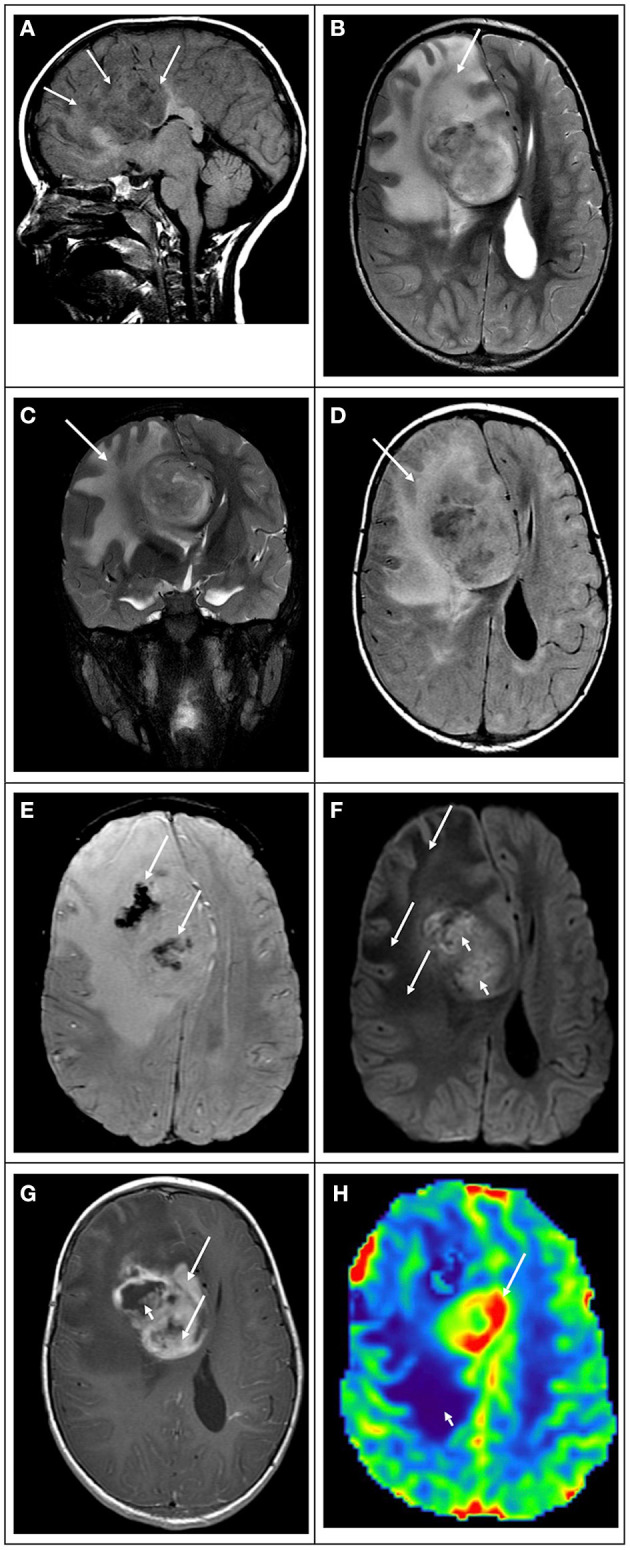
5-year-old male with headaches and confusion due to a right frontal “glioblastoma.” **(A)** T1 weighted image in the sagittal plane shows that the lesion is heterogeneous, ill-defined with mixed-signal, predominantly hypointense, and infiltrates the corpus callosum (white arrows). **(B)** T2 weighted image in the axial plane, **(C)** T2 weighted image in the coronal plane, and **(D)** FLAIR image in the axial plane show that the bulk of the lesion is hyperintense, with marked mass effect and vasogenic edema (white arrows). **(E)** Susceptibility weighted image in the axial plane shows irregular areas of signal drop within the tumoral bed (white arrows) in keeping with hemorrhage. **(F)** Diffusion-weighted image in the axial plane shows that the lesion is heterogeneous in signal. The extensive area of vasogenic edema shows low signals (white arrows). The midline component shows areas of hyperintense signal with low values in the ADC map (not shown) in keeping with restricted diffusion. **(G)** T1-weighted contrast-enhanced image in the axial plane shows that the tumor enhances heterogeneously. The midline component of the mass shows thick and irregular enhancement (white arrows) with no foci of enhancement in areas of necrosis (white arrowhead). **(H)** Arterial spin labeling perfusion image in the axial plane shows that the midline component has marked increased perfusion (white arrow). Note that the area of vasogenic edema is not associated with increased perfusion (white arrowhead).

In general terms, most HGGs typically show higher perfusion (increased CBV and/or CBF) than low-grade gliomas (Figure 10). Nevertheless, compared to adults, the higher prevalence in children of malignant non-glioma neoplasms and contrast-enhancing low-grade tumors may confound the accuracy of grading of brain neoplasms using PWI measures in certain types of neoplasms.

#### Dynamic Susceptibility Contrast (DSC) Perfusion

DSC perfusion is the most widely used PWI technique and requires high-flow contrast injection, often using power injectors, and large-bore intravenous access, which may pose challenges in young children and infants (95). Other drawbacks of the DSC perfusion method are calcification and hemorrhage-induced susceptibility within the tumor and blood-brain barrier breakdown-related contrast leakage (96). Despite this, DSC is feasible and has been performed even in young children (97).

There is an overall significant difference in rCBV and CBF between pediatric low-grade gliomas and HGGs (98). In addition, in the study by Chang et al. (35), rCBV maps helped detect highly vascular areas correlated with areas of enhancement in three of their patients. rCBV maps may also demonstrate increased microvascularity even in the absence of enhancement in some patients with “glioblastoma” (35).

#### Dynamic Contrast Enhancement (DCE)

DCE may be used as a potential alternative or complementary technique to DSC. It has been mainly used in adult brain tumors, with few studies performed in children (99, 100). DCE provides signal intensity–time curve reflecting a combination of tissue perfusion, microvessel permeability, and extravascular-extracellular space characteristics (101, 102) thus allowing for a multiparametric characterization of tumor microvasculature and leakage quantitation.

The advantages of DCE over DSC are fewer susceptibility artifacts and the quantification of blood-brain barrier (BBB) integrity; indeed, the leading interest for DCE-derived metrics was initially focused on the volume transfer constant (Ktrans), a permeability marker correlating with BBB disruption (102) and malignancy (103). Conversely, DSC typically offers better temporal resolution than DCE, allowing potentially better blood volume estimation (104).

A small study demonstrated that DCE could be used to assess tumor grade in pediatric brain tumors, although not all were gliomas (100). Transfer constants from and into blood plasma (K_trans_ and K_ep_) and extracellular extravascular volume fraction (V_e_) showed a sensitivity of 71–76% and a specificity of 82–100% in separating low-grade from high-grade tumors. In another study, fractional plasma volume (V_p_) was significantly different between high and low-grade tumors, but K_trans_, K_ep_, and V_e_ were not statistically different.

In a study of 64 pediatric brain tumor patients, Gupta et al. (105) demonstrated that DSC and DCE helped differentiate low-grade tumors, high-grade tumors, and amongst major posterior fossa tumors. rCBV and fractional plasma volume measures differed significantly between high-grade and low-grade tumors. High-grade tumors could be differentiated from low-grade tumors with an rCBV cutoff value of 2.41 and 88.6% sensitivity and 65% specificity. There was no significant difference in Ktrans, Kep, or Ve between these two groups of tumors (105).

#### Arterial Spin Labeling (ASL) Perfusion

ASL, a PWI technique that uses magnetically labeled water as endogenous contrast, has been used to study pediatric brain tumors (106). However, ASL is limited by a low signal-to-noise ratio, often the need for greater magnetic field strength, and the presence of susceptibility (105).

ASL is considered a reliable PWI technique in evaluating tumor perfusion and predicting glioma grade in adults (107). ASL is advantageous for children since it lacks contrast injection, the need for high flow injections, and has easier potential for CBF quantification. Also, ASL can be repeated if patients move (95). Moreover, younger children's immature paranasal sinuses also result in better ASL image quality, with potentially lesser degrees of distortion artifacts in the frontal and inferior brain regions (108).

In a study of ASL in pediatric brain tumors by Yeom et al. (95), the authors demonstrated that the technique could reasonably distinguish high-grade from low-grade tumors. Their results in “glioblastoma” patients indicated that CBF in the tumoral bed might have a wide range, which suggests vascular heterogeneity similar to what is seen in patients with adult glioblastomas. According to these authors, ASL maps can depict tumor vascular heterogeneity and indicate higher tumor blood flow regions offering a valuable parameter to potentially direct biopsy of higher vascular density or more malignant regions (95). Dangouloff-Ros et al. (106) have confirmed that pediatric high-grade brain tumors generally display higher CBF than low-grade tumors on ASL. Low-grade gliomas had a significantly lower absolute CBF and rCBF than high-grade tumors (CBF: median, 29 mL/min/100g vs. median, 116 mL/min/100 g; *P* < 0.001) (rCBF: median, 0.50 mL/min/100 g vs. median, 2.21 mL/min/100 g; *p* < 0.001). There was no significant difference between the various high-grade neoplasms (grade 3 gangliogliomas, glioblastomas, atypical teratoid rhabdoid tumor, and grade 3 ependymomas) (106). Morana et al. (109) compared ASL and DSC in 37 children with low-grade and HGGs obtained on a 1.5T scanner. Normalized CBV values in the most perfused area of each neoplasm were compared with normalized CBF from DSC and normalized CBF from ASL data and designated with a WHO tumor grade. According to the authors, normalized ASL provides comparable results to DSC and may help distinguish between low-grade and HGGs (109).

A meta-analysis of eight studies assessing pediatric glioma grading using ASL showed many bias and applicability issues. For low and high-grade tumor differentiation, the pooled sensitivity ranged from 0.69 to 0.92, and specificity ranged from 0.63 to 0.93 (110). Relative CBF demonstrated less variability than absolute CBF, as would be expected given the variability of acquisition techniques. In another meta-analysis, although not only composed of gliomas, normalized cerebral blood flow derived from ASL perfusion had 83% accuracy in separating low and high-grade pediatric brain tumors (111). Other authors have also confirmed that ASL can be a potentially valuable tool to differentiate low from high-grade tumors (106, 111–114) and that the technique has comparable results with DSC in the differentiation between low and HGGs (115).

### Proton ^1^H Magnetic Resonance Spectroscopy

Proton ^1^H MRS is a non-invasive technique that has been used to evaluate tissue metabolism in a wide variety of diffuse and focal CNS diseases, including brain tumors (116). Several brain metabolites such as choline (Cho), N-acetylaspartate (NAA), creatine (Cr), myoinositol (mI), and lactate can show abnormalities in the context of brain neoplasms. For example, in gliomas, as tumor grade increases, there is an increase in the Cho/NAA ratio, reflecting increased metabolic activity. High Cho reflects rapid membrane turnover with glial proliferation, whereas low NAA reflects decreased normal neurons as they are replaced by neoplastic cells in the MRS voxel (35).

**Figure 11:**
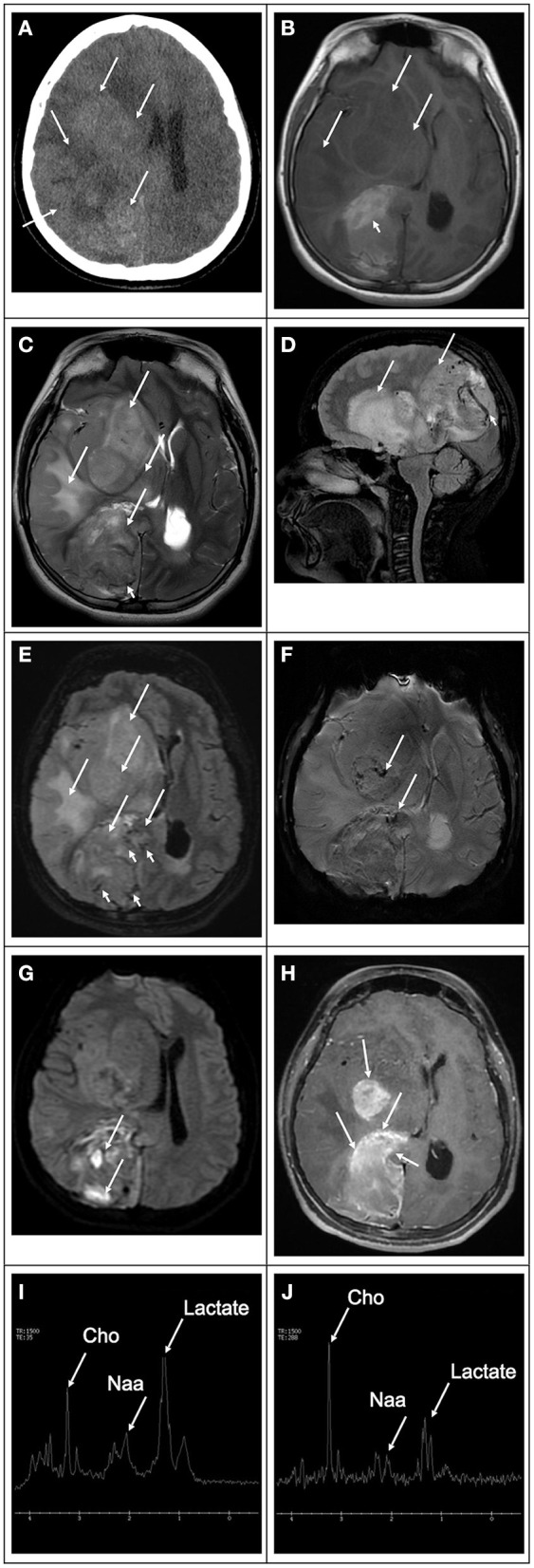
8-year-old- female with a hemispheric glioblastoma. **(A)** Computed tomography image in the axial plane shows an extensive mass in the right hemisphere (white arrows), causing mass effect, compression of the right lateral ventricle, and midline deviation. The lesion is heterogeneous with areas of hyper and hypodensity compatible with hemorrhage and edema, respectively. **(B)** T1 weighted image in the axial plane shows that the bulk of the lesion is hypointense (white arrows). There is a component of the mass that is high in signal intratumoral hemorrhage (white arrowhead). **(C)** T2 weighted image in the axial plane. **(D)** T2 weighted image in the sagittal plane, and **(E)** FLAIR image in the axial plane show that the lesion's bulk is hyperintense with marked mass effect vasogenic edema (white arrows). In addition, there is marked increased vascularity in the component located in the occipital region (white arrowheads). **(F)** Susceptibility weighted image in the axial plane shows scattered signal drop foci within the tumoral bed (white arrows) in keeping with hemorrhage. **(G)** Diffusion-weighted image in the axial plane shows that the lesion is heterogeneous in signal. The bulk of the lesion is isointense to the normal brain parenchyma with scattered hyperintense foci due to intratumoral hemorrhage (white arrows). **(H)** T1-weighted contrast-enhanced image in the axial plane shows that the tumor has multiple enhancing components heterogeneously. The mass has avid peripheral enhancement with internal no enhancing elements in keeping with necrotic tissue. **(I,J)** Magnetic resonance spectroscopy obtained with short and long echo times showing marked decreased N-acetylaspartate peak, which corresponds to neuronal loss, increased choline peak, which corresponds to increase cell membrane turnover in an environment of rapid growing neoplastic tissue, and increased lactate peaks due to anaerobiosis and necrotic changes.

Multiple studies have demonstrated that a common feature of many rapidly growing tumors is a decreased NAA/Cr ratio and an increased lactate level (Figure 11) (37, 117–122). Cr concentration is supposedly rather stable over time, so it is used as a denominator to report the metabolite ratios in most studies. Elevated lipid-lactate peaks are more frequently found in HGGs, particularly in those undergoing central necrotic changes. Mixed MRI spectral patterns can reflect the known heterogeneity seen in many tumors (123). Importantly, patients with diffuse midline gliomas showing high levels of lactate on MRS are associated with poor prognosis (124).

Proton ^1^H MRS may be used as an adjunct tool in the evaluation of “pediatric glioblastomas.” This technique has been applied for the initial diagnosis of brain masses, biopsy guidance, tumor grading, treatment response assessment, recurrence vs. treatment effects detection, and a prognostic marker in brain tumor patients. Higher Cho/Cr and Cho/NAA ratios are typically observed in HGGs compared to low-grade tumors and non-neoplastic masses in broad terms, although exceptions do occur (125). mI is often elevated in low-grade diffuse gliomas, unlike HGGs (123). Previous studies have shown that higher Cho/Cr and Cho/NAA ratios portray a poorer prognosis in pediatric brain tumors (126, 127). Other studies have looked at lactate/NAA ratios and normalized indices combining choline and lipid+lactate levels, demonstrating strong correlations with predictors of outcome (128, 129).

Pathogenic variants in the IDH1 and IDH2 can lead to the accumulation of abnormally high D-2-hydroxyglutarate (2-HG) levels in certain brain tumors. This increased 2-HG level may be detected *in-vivo* by specialized advanced spectral-edited MRS and used to characterize glial neoplasms. IDH1/2 pathogenic variants are considered prognostic biomarkers in subjects with glioma and are associated with more prolonged overall survival (24, 130). A meta-analysis demonstrated excellent sensitivity and specificity of 2-HG MRS to predict *IDH* mutant gliomas (131).

### Chemical Exchange Saturation Transfer

Chemical exchange saturation transfer (CEST) is a recently developed technique that can identify very low concentrations of molecules by the presence of groups with exchangeable protons, such as hydroxyls, amides, and amines (132). The vast majority of studies on CEST and “glioblastomas” have been performed in adults. Future studies on “pediatric glioblastomas” are needed to confirm its utility in this age group. CEST signal is affected by endogenous proteins and metabolites such as glutamate, lactate, mI, and glucose that play crucial roles in tumor development, growth, and progression. Hence, studying these macromolecules and metabolites may help understand the brain tumor microenvironment and evaluate response to targeted therapies. Studies have demonstrated that CEST imaging can reasonably differentiate malignant neoplastic infiltration from peritumoral vasogenic edema, differentiate histopathological grades, and discriminate HGGs from the brain lymphomas (133). In addition, CEST can also reliably distinguish recurrent tumors from radiation necrosis (133). Moreover, the intensity of the CEST signal was shown to decrease in irradiated tumors at 3 days and 6 days post-treatment periods relative to baseline, suggesting that CEST may potentially help evaluate treatment response in brain tumors. CEST signal may potentially be an imaging biomarker for distinguishing true progression from pseudoprogression in “glioblastoma” patients (134).

Amide proton transfer (APT) imaging is a CEST technique that measures a decrease in bulk water intensity due to chemical exchange with magnetically labeled amide protons of endogenous proteins and peptides in tissues (135). Studies have shown the potential of APT-weighted imaging in delineating malignant neoplastic infiltration. In addition, the APT signal may also potentially be a valuable imaging biomarker for distinguishing true progression from pseudoprogression in glioblastoma patients (133). Studies have documented significantly higher APT signals in true progression cases than those with pseudoprogression (136). It is widely believed that active tumor cells express more protein species and higher concentrations of protein components, as shown by proteomics (136, 137) and proton MRS studies (138). In contrast to true progression, there are fewer mobile cytosolic proteins and peptides in regions of brain injury associated with pseudoprogression due to lower cellular density and disrupted cytoplasm. Collectively, these studies have indicated that APT based on CEST is fast emerging as a novel molecular MRI technique in neuro-oncology.

### Multiparametric Analysis

Multiparametric analysis is an evolving method in which multiple quantitative MRI techniques are analyzed in combination to overcome the intrinsic limitations of conventional MRI and potentiate the individual value of each advanced MRI technique in isolation. For example, quantitative data obtained from metabolic and physiologic techniques such as DWI, DTI, DSC, DCE, ASL, or proton MRS can be variously combined and analyzed with multivariate logistic regression, analysis of variance, or artificial intelligence tools to determine the optimal parameter(s) and thresholds for addressing a specific question prediction.

Multiparametric MRI has been widely used to predict treatment response in glioblastoma patients, to differentiate glioblastoma from solitary brain metastasis (139), to differentiate radiation necrosis from a recurrent tumor (140), to assess tumor invasiveness (141), and to predict tumoral survival (142).

## Positron Emission Tomography (PET)

Positron emission tomography (PET) is a nuclear medicine imaging modality that plays a vital role in evaluating brain tumors, including glioblastomas. PET, as a functional imaging technique, is typically used to complement anatomical imaging, capturing non-invasively functional and biochemical information about the tumor and its surroundings. This information is beneficial for tumor grading, differential diagnosis, tumor delineation, surgery planning, radiotherapy, and post-treatment monitoring follow-up (143). PET can probe the physiological milieu of neoplastic cells, such as glucose metabolism, protein synthesis, and DNA replication. There are multiple groups of PET radionuclide tracers typically used to evaluate tumor metabolism, including glucose metabolism tracers, amino acid tracers, and other miscellaneous tracers that can target various receptors or functions of the cells. These PET tracers are useful in demonstrating cellular proliferation, hypoxia sensing, and inflammation (144).

A typical and widely used glucose metabolism tracer is 18F-2-fluoro-2-deoxy-D-glucose ([18F]FDG). [18F]FDG is taken up by the glucose transporter, engaging in phosphorylation (the first step of glucose metabolism), and subsequently becomes trapped in the cell (145). [18F]FDG demonstrates avid uptake in the brain, which may limit the interpretation of tumors near or involving the gray matter (144). On the other hand, amino acid transport tracers exhibit lower uptake in normal tissue, being more sensitive than 18F-FDG in primary and recurrent tumors, and are helpful in differentiating recurrent tumors from treatment-induced changes (146). Typical amino acid analogs include [11C]11C-methyl-methionine ([11C]MET), 3,4-dihydroxy-6-[18F]-fluoro-L-phenylalanine ([18F]FDOPA), O-(2-[18F]-fluoroethyl)-L-tyrosine ([18F]FET), and deoxynucleoside bases such as [18F]fluoro-thymidine and [18F]clofarabine (143, 145).

[11C]MET is the most studied and validated amino acid tracer. It is an essential amino acid for protein synthesis and is considered more sensitive than [18F]FDG in delineation of tumors. [11C]MET is avidly taken up by glioma cells, with only a low uptake in normal cerebral tissue. Several studies have suggested that tumor uptake of [11C]MET mainly reflects increased amino acid transport (147). Its most important limitation is its very short life (144). 18F-labeled amino acids have been created to increase amino acid tracer half-life and utilization, namely [18F]FET and [18F]FDOPA. [18F]FET is a tracer for both HGGs as well as low-grade gliomas due to an efficient nucleophilic reaction, elevated uptake by tumor tissues, low uptake by inflammatory tissues, and high stability (148). [18F]FDOPA is incorporated into cells through amino acid transporters that are overexpressed in gliomas, and its transport and uptake are independent of the blood-brain barrier (149). [18F]FDOPA has shown a significant correlation between WHO grade and the volume of MRI contrast enhancement and volume of T2 hyperintensity (150). Another radiotracer, [18F]fluorothymidine ([18F]FLT), is a thymidine analog uptaken in tissues after phosphorylation by the thymidine kinase (151). Thymidine kinase is considered a critical enzyme in the DNA salvage pathway. Nucleotide salvage pathways recover bases and nucleosides that are formed during the degradation of RNA and DNA (152). Since [18F]FLT has lower uptake in the normal brain, it correlates well with proliferative tissue markers (153). The radiotracer has been used in diagnosis and assessment of glioma grading, in differentiating tumor recurrence from radionecrosis, in assessing response to treatment, and in predicting overall survival (154).

PET tracers such as the [18F]Fluoromisonidazole, which sense oxygen levels in cells, can be used to visualize hypoxia. Hypoxia is an essential feature of most solid tumors (153, 155). For example, in a hypoxic tumor microenvironment, radiation therapy could be more effective at a higher dose (143). Inflammation, a component of the immune response, is well-known to occur in glioblastomas. Translocator protein (TSPO) is a component of the mitochondrial membrane protein responsible for cholesterol transport and responds to cell stress. Broadly, TSPO is treated as a biomarker sensitive to pro-inflammatory stimuli (143). Moreover, TSPO PET imaging was found to be correlated with outcome (156).

As neuro-oncology treatment advances and therapies centered on tumor biomarkers are discovered, the development of selective PET tracers can dramatically enhance the efforts toward personalized medicine (143).

## Treatment

The standard of care for adult glioblastomas involves surgery with maximal safe tumor resection followed by chemoradiation as adjuvant treatment (157). There is not such a well-defined standard of care for “pediatric glioblastomas.” Regarding surgery, substantial evidence recommends the adoption of maximal safe surgical resection of the visible tumor, since prognosis correlates positively with the amount of tumor resected (5, 6, 16, 158, 159). However, the choice of adjuvant treatment is debated due to the potentially deleterious effects of radiation treatment in the early developing brain and inconsistent results of various chemotherapy regimens (9). Radiation therapy has become the standard of treatment, particularly for those children older than 3 years with a newly diagnosed “glioblastoma” (36). Younger children, however, are more susceptible to the adverse effects associated with radiation therapy and are typically treated with chemotherapy alone and radiation-sparing approaches (160–162).

Novel classes of treatments such as molecular-targeted therapy and immunotherapy have been recently incorporated as new weapons in the arsenal of treatment of oncology patients (163). Immunotherapy agents include typically chimeric antigen receptor (CAR) T-cell therapy, immune checkpoint inhibitors, virotherapy, cancer vaccines, and dendritic cell therapy. Molecular-targeted therapy consists of drugs or other substances that target specific molecules involved in the growth and spread of cancer cells (164). The most common targets for molecular-targeted therapy in pediatric brain tumors, in particular, HGGs and other high-grade tumors, are the tyrosine kinase (165), tumor-specific surface proteins (monoclonal antibodies) (166), vascular endothelial growth factor (167), platelet-derived growth factor and epidermal growth factor, PI3K-mTOR pathway (mostly animal model studies) (168), ERK-RAS-RAF mitogen-activated protein kinase pathway (BRAF mutation) (169), e PI3K/AKT pathway, gene fusions (170), Sonic Hedgehog pathway (171), and epigenetic targets (DNA methylation, chromatin modeling, and histone modifications signaling) (172). Despite multiple clinical trials in pediatric brain tumors, molecular-targeted therapy, with some exceptions, has not yet made a major impact on survival or, for that matter, quality-of-life for children with brain tumors (164). However, there remains great promise for the future as more agents are developed and included in clinical trials.

CARs are synthetic receptors composed of three major components (an extracellular tumor-specific antibody, an intracellular signaling structure, and a transmembrane domain serving as a bridge), which are typically added to a T cell, augmenting its function (173–175). These receptors allow T cells to recognize and destroy specific cancer cells without the major histocompatibility complex (MHC) presentation (176). In normal conditions, T cells require costimulation by the antigen-presenting cell to exert their cytolytic activity (177). This method has had tremendous success in treating leukemias. CAR T cell administration can be made hematogenously, or via the cerebral spinal fluid, or locally in the tumor cavity. Brain tumors are currently one of the most common solid tumor types undergoing clinical trial testing for CAR T cell efficacy and have shown early promise in treating “glioblastomas” (178, 179). Multiple clinical trials are evaluating the efficacy of CAR T cell therapy in solid tumors. However, none are FDA approved yet.

Checkpoint inhibitors are a group of drugs that target signaling pathways involved in the modulation of host immune responses as part of the normal regulation of immunity and establishment of tolerance. However, neoplastic cells are known to block these checkpoints and suppress the host's immune response, bypassing immune recognition and destruction (180). Exhaustion is a phenomenon that occurs during cancer progression, which refers to immune cell desensitization. At exhaustion, T cells are unable to kill malignant cells (181, 182), which can occur when neoplastic cells upregulate inhibitory receptors such as programmed cell death receptor-1 ligand (PD-L1) and cytotoxic T lymphocyte antigen-4 (CTLA-4) (183). Checkpoint inhibitors seek to reverse this inhibition so that an immune response can be mounted against the malignant cells (180). Researchers have shown that 75–100% of gliomas in a sample of patients exhibited PD-L1 expression, which correlated with the severity of the disease (184, 185).

There are several studies describing the efficacy and side effect profile of checkpoint inhibitors in pediatric patients. A clinical trial conducted by Gorsi et al. (186) in recurrent or refractory pediatric brain tumors treated with the immune checkpoint inhibitor nivolumab demonstrated transient partial responses in patients with positive PD-L1 expression and higher tumor mutation burden. Nivolumab was well-tolerated with some transient partial responses in patients with positive PD-L1 expression and a higher tumor mutation burden. Median survival for PD-L1 positive patients was 13.7 weeks vs. 4.2 weeks for PD-L1 negative patients (ρ = 0.08). These findings suggest that only tumors with elevated PD-L1 expression and tumor mutation burden may benefit from immune checkpoint inhibitors (186). A clinical trial by Cacciotti et al. (187) involving PD-1 and CTLA-4 blockade has shown relatively well tolerability in a group of pediatric patients with DIPG, HGG, ependymoma, craniopharyngioma, high-grade neuroepithelial tumor, and non-germinomatous germ cell tumor. The majority of the patients, during the time of the study, showed stable disease or partial response. Checkpoint inhibition has proved to be a promising new form of immunotherapy, demonstrating efficacy in adult malignancies such as melanoma, renal cell carcinoma, and small cell lung cancer; however, it has failed to produce durable responses in pediatric brain cancer (188).

Virotherapy is an emerging technique that uses viruses as therapeutic agents. Oncolytic viruses combine tumor cell destruction and immune system stimulation, which are the most critical components of virotherapy (189). In addition, viruses can also be used as vectors for gene therapy, inducing the expression of a transgene that modifies the immune environment to promote an anti-tumor response (189). Several oncolytic viruses have been evaluated clinically in pediatric brain tumors, such as myxoma virus (190, 191), recombinant poliovirus (192, 193), adenovirus (194), Seneca Valley-001 (195), reovirus (196), herpesvirus (197), and Newcastle disease virus (198). The administration of oncolytic viruses can be either intravenous or intratumoral. Once the virus reaches the tumor, it can infect both normal and neoplastic cells; however, it only replicates and destroys the tumor cells (198). Cancer vaccines are typically well-tolerated and contain tumor antigens, such as peptides, tumor lysate, nucleic acids, and autologous dendritic cells (196, 199). Mutations such as the H3 K27M in DIPG have been explored as a target for peptide vaccines (199). A number of clinical trials are evaluating the efficacy of virotherapy in pediatric brain tumors. However, none are FDA approved yet.

Dendritic cells are an essential link between the innate and adaptive immune systems. Upon finding foreign antigens, dendritic cells release inflammatory cytokines that activate the immune system. These cells also process and present antigens to T cells and B cells, thereby activating naïve, effector, and memory immune cells or maintaining tolerance against self-antigens (200). For active immunotherapy, dendritic cells are typically generated by isolating monocytes from cancer patients that are expanded and activated *ex vivo*. These cells are loaded with either tumor lysate, peptides, nucleic acids, or viral epitopes that are expressed by the tumor. Dendritic cells are usually matured with GM-CSF, a critical cytokine, then administered as a vaccine. Adjuvants such as tetanus toxoid are important to improve inflammation and immunogenicity in the host (200). Dendritic cell vaccines have demonstrated modest but encouraging results in patients with advanced cancers (201). It is thought that these vaccines can induce tumor-specific T cell responses and immunological memory, constituting a promising platform for pediatric brain tumors (201). There are several trials using dendritic cell vaccines and tumor RNA (201) or tumor lysate for pediatric brain tumors (202, 203). Dendritic cell vaccines are reliably manufactured and extremely well-tolerated; however, for efficacy improvement, enhanced techniques for targeting, antigen loading, and migration *in vivo* are needed (196). There are a number of dendritic cell vaccines in clinical trials; none are available for widespread clinical use.

Immunotherapy has shown efficacy against lymphoid tumors and some solid neoplasms, with less efficacy in the treatment of pediatric brain tumors. Reasons for this limited response include tumor heterogeneity, a suppressive immune microenvironment, and the blood-brain barrier (163). In addition, most pediatric brain tumors are immunologically quiescent, with a low mutational burden. Therefore, immunotherapy strategies should be tailored based on the type of tumor being targeted and its associated microenvironment (163). According to the National Cancer Institute at the National Institutes of Health, there are only six FDA-approved immunotherapy options for brain and nervous system tumors, namely dostarlimab, a checkpoint inhibitor that targets the PD-1/PD-L1 pathway for patients with advanced cases associated with DNA mismatch repair deficiency; granulocyte-macrophage colony-stimulating factor, an immunomodulatory cytokine combined with naxitamab-gqgk, for advanced cases of neuroblastoma; pembrolizumab, a checkpoint inhibitor that targets the PD-1/PD-L1 pathway for patients with advanced cases associated with high microsatellite instability, DNA mismatch repair deficiency, or high tumor mutational burden; bevacizumab, a monoclonal antibody that targets the VEGF/VEGFR pathway and inhibits tumor blood vessel growth for advanced “glioblastoma”; dinutuximab, a monoclonal antibody that targets the GD2 pathway for first-line treatment of high-risk pediatric neuroblastoma; naxitamab-gqgk, a monoclonal antibody that targets the GD2 pathway and approved in combination with GM-CSF for a subset of patients with advanced neuroblastoma.

Regardless of the treatment selected, “pediatric glioblastoma” remains a devastating disease, with median survival ranging from 13 to 73 months, with a 5 year survival of <20% (4–6, 204). Nevertheless, several studies have demonstrated a relatively better prognosis and long-term survival among pediatric patients compared to adults (5–7).

## Final Remarks

Glioblastomas are highly aggressive neoplasms that represent a small subset of pediatric brain tumors. “Pediatric glioblastomas” are grossly and microscopically identical to their adult counterparts, but molecularly and genetically distinct. These differences may explain why “pediatric glioblastomas” may have a different prognosis or occasionally be less responsive to current adjuvant therapy regimens.

MRI is the imaging modality of choice in evaluating “pediatric glioblastomas,” not only because it is safer due to the absence of radiation exposure but also for its superior spatial and contrast resolution and for delivering physiologic information about the tumor and adjacent brain parenchyma. MRI is also vital for surgical and radiation therapy planning and postsurgical evaluation. Conventional MRI sequences provide precise anatomic detail and detection of blood-brain barrier integrity and leakage. Advanced MRI techniques such as DWI, PWI, and proton ^1^H MRS may help differentiate non-enhancing tumors from other causes of changes in the signal. It is expected that continued research increases the availability of advanced MRI techniques. This increment, coupled with emerging MRI techniques and advances in personalized medical care, perhaps powered by artificial intelligence techniques, would hopefully result in a better quality of life and overall improvement in survival rates.

## Author Contributions

All authors listed have made a substantial, direct and intellectual contribution to the work, and approved it for publication.

## Conflict of Interest

The authors declare that the research was conducted in the absence of any commercial or financial relationships that could be construed as a potential conflict of interest.

## Publisher's Note

All claims expressed in this article are solely those of the authors and do not necessarily represent those of their affiliated organizations, or those of the publisher, the editors and the reviewers. Any product that may be evaluated in this article, or claim that may be made by its manufacturer, is not guaranteed or endorsed by the publisher.
